# Supplementary material for: Genome-Wide Identification and In Silico Analysis of ZF-HD Transcription Factor Genes in Zea mays L
Source: Genes (Basel). 2022 Nov 14;13(11):2112. doi: 10.3390/genes13112112 (PMC9690586; doi:10.3390/genes13112112)
Supplement: Supplementary file 1 [file genes-13-02112-s001.zip › genes-2009463-Supplementary File S1.pdf]

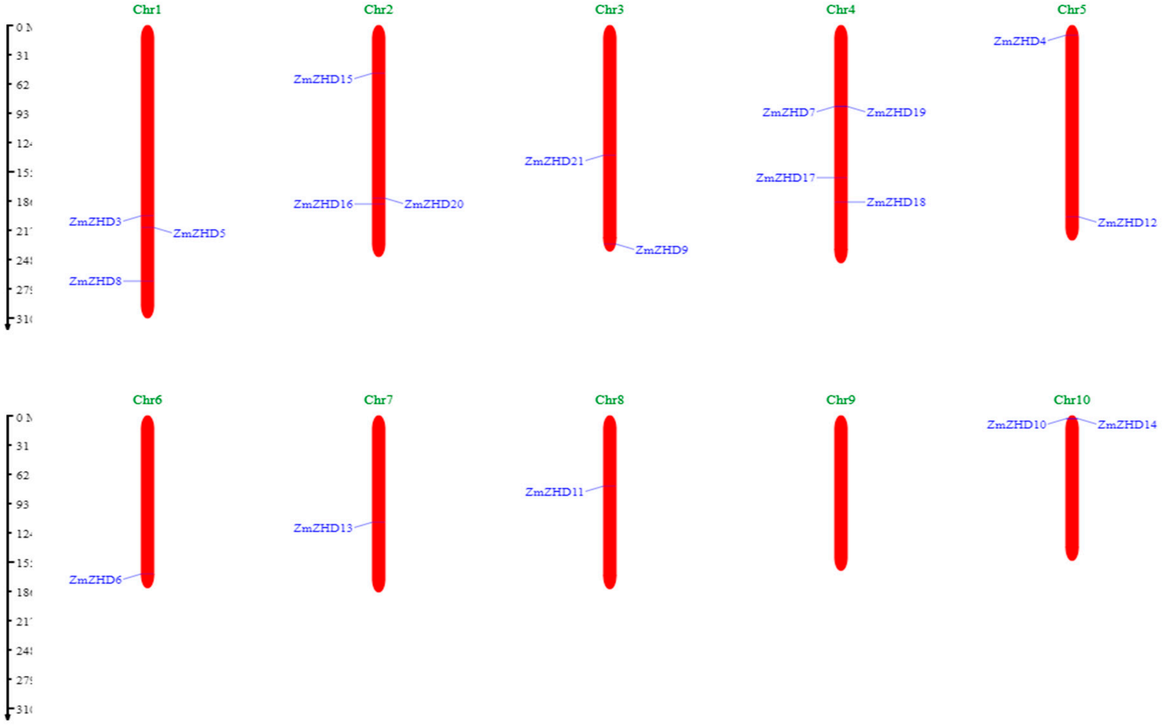

Supplementary Figure S1: Chromosomal location of the ZmZHD genes in the 10 chromosomes of *Zea mays*

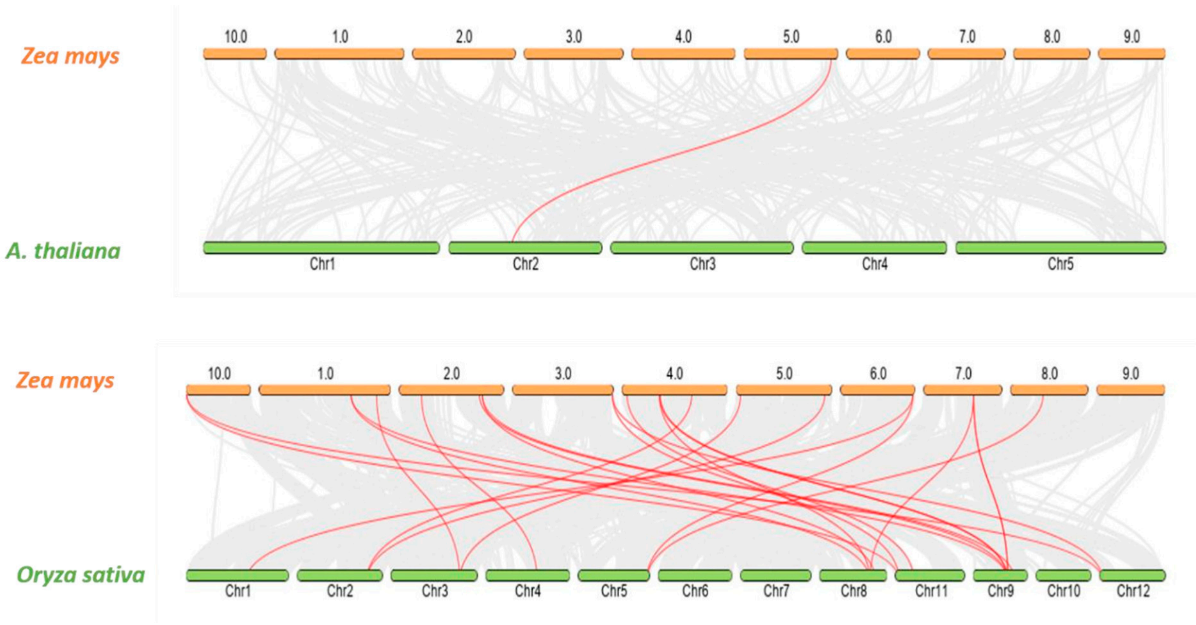

Figure S2: Synteny analysis of the maize genome with *A. thaliana* and *Oryza sativa*.

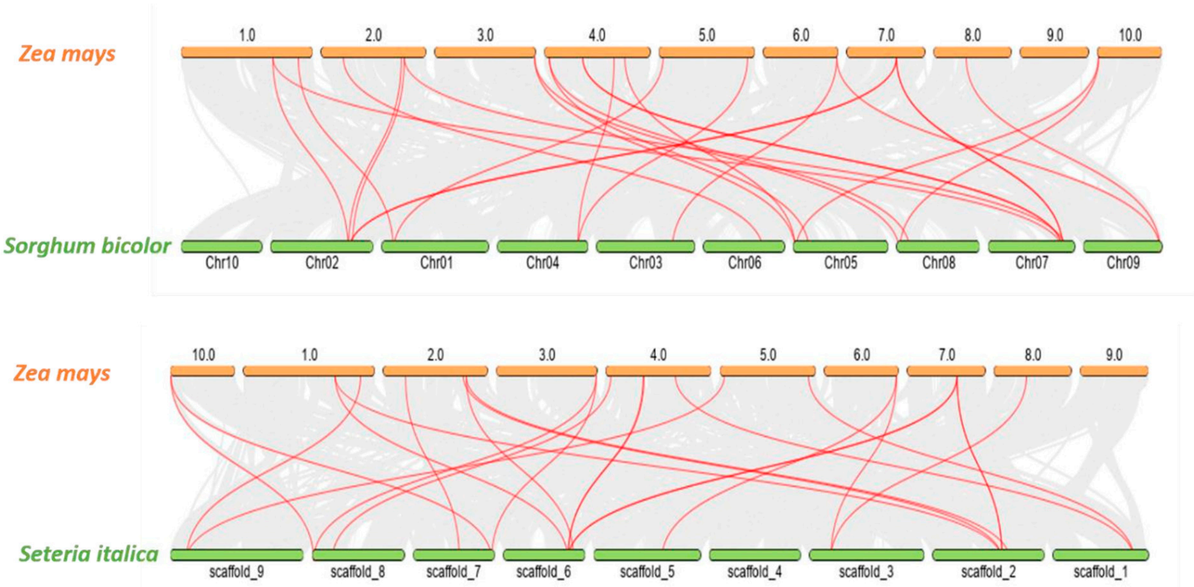

Figure S3: Synteny analysis of the maize genome with *S. bicolor* and *S. italic*.
